# Engineering Bispecificity into a Single Albumin-Binding Domain

**DOI:** 10.1371/journal.pone.0025791

**Published:** 2011-10-03

**Authors:** Johan Nilvebrant, Tove Alm, Sophia Hober, John Löfblom

**Affiliations:** 1 Department of Proteomics, School of Biotechnology, Royal Institute of Technology, AlbaNova University Center, Stockholm, Sweden; 2 Department of Molecular Biotechnology, School of Biotechnology, Royal Institute of Technology, AlbaNova University Center, Stockholm, Sweden; Deutsches Krebsforschungszentrum, Germany

## Abstract

Bispecific antibodies as well as non-immunoglobulin based bispecific affinity proteins are considered to have a very high potential in future biotherapeutic applications. In this study, we report on a novel approach for generation of extremely small bispecific proteins comprised of only a single structural domain. Binding to tumor necrosis factor-α (TNF-α) was engineered into an albumin-binding domain while still retaining the original affinity for albumin, resulting in a bispecific protein composed of merely 46 amino acids. By diversification of the non albumin-binding side of the three-helix bundle domain, followed by display of the resulting library on phage particles, bispecific single-domain proteins were isolated using selections with TNF-α as target. Moreover, based on the obtained sequences from the phage selection, a second-generation library was designed in order to further increase the affinity of the bispecific candidates. Staphylococcal surface display was employed for the affinity maturation, enabling efficient isolation of improved binders as well as multiparameter-based sortings with both TNF-α and albumin as targets in the same selection cycle. Isolated variants were sequenced and the binding to albumin and TNF-α was analyzed. This analysis revealed an affinity for TNF-α below 5 nM for the strongest binders. From the multiparameter sorting that simultaneously targeted TNF-α and albumin, several bispecific candidates were isolated with high affinity to both antigens, suggesting that cell display in combination with fluorescence activated cell sorting is a suitable technology for engineering of bispecificity. To our knowledge, the new binders represent the smallest engineered bispecific proteins reported so far. Possibilities and challenges as well as potential future applications of this novel strategy are discussed.

## Introduction

Monoclonal antibodies have been widely used in nearly all areas of life science for over three decades and represent a growing class of agents also in the clinics, mainly due to their generally high specificity and excellent pharmacokinetic properties. Today, regulatory authorities have approved over 20 monoclonal antibodies for therapeutic or diagnostic use [1], [2]. In addition to full-length monoclonal antibodies, smaller antibody derivatives (e.g. scFvs and Fab fragments) as well as entirely new protein architectures [3], [4], [5] have been investigated for similar purposes. The non-immunoglobulin based affinity proteins are in general derived from single domain scaffolds with attractive biophysical properties, such as high stability and solubility. The size of these alternative scaffolds is typically smaller compared to antibodies, which provides means for cost-efficient production in bacteria. For the smallest scaffolds there is also a possibility to use solid-phase peptide synthesis for production, enabling site-specific conjugation of non-biological groups (e.g. chelators and payloads) as well as engineering of new physicochemical properties into the agent [6]. For molecular imaging (e.g. in cancer prognostic and diagnostic applications), the reduced size of such alternative scaffolds generally results in an improved tumor-to-blood contrast due to the rapid tumor penetration and *in vivo* clearance rate [7], [8], [9]. Furthermore, the small size and straightforward recombinant manipulation make alternative affinity proteins an excellent choice for generation of bi- and multispecific molecules [10]. Several of the reported alternative scaffolds are also based on domains that are found as repetitive elements in natural proteins (e.g. affibody molecules that are originally derived from protein A [11]), supporting the strategy of using them in bi- and multispecific constructs. However, although fusing such domains into multispecific chains is relatively straightforward, it also has an impact on the overall size of the final molecule, which might negatively influence some of the favorable properties.

In this study, we take the concept of bispecific affinity proteins one step further by engineering the specific targeting directly into an albumin-binding domain, thus creating a single-domain bispecific affinity protein. In order to engineer such a small protein domain with dual affinities, an alkali-stabilized variant [12] of a natural albumin-binding domain (ABD) was chosen as scaffold. ABD is a 46 amino acid, three-helical bundle protein [13] with the albumin-binding site mainly in the second helix [14], [15], [16]. Hence, eleven amino acids in helix one and three were chosen for randomization and the resulting library was displayed on phage particles for subsequent selections [17]. In this project, the objective was to select bispecific binders that were able to interact with TNF-α, and still retain the affinity towards human serum albumin (HSA). Binding to albumin in the blood through fusion of the molecule of interest to albumin-binding domains has been shown to provide substantial half-life extensions for various recombinant proteins *in vivo* [18], [19]. TNF-α is a pleiotropic proinflammatory cytokine that plays an important pathogenic role, particularly in inflammatory disorders [20]. Both the cell-associated and the secreted forms of TNF-α are biologically active in the form of homotrimers and inhibition of TNF-α has proven to be an effective therapy for a number of inflammatory diseases (e.g. rheumatoid arthritis). Several protein-based antagonists are currently approved for clinical use [2]. Engineering TNF-α-binding into a small protein with an intrinsic extended half-life may reduce the dosing frequency and thereby the associated treatment cost. This strategy, hence, aims to combine the advantages of small size (e.g. efficient production in a bacterial host or by chemical synthesis) with the long persistence in the circulation that is typical for antibodies.

In order to succeed with the challenging effort of engineering such bispecific affinity proteins, phage display was used for selection of first-generation TNF-α specific binders from the combinatorial ABD-library. In the following lead optimization, cell display in combination with fluorescence activated cell sorting (FACS) was utilized for affinity maturation as well as efficient multiparameter sorting of bispecific variants that showed strong binding to TNF-α while still retaining their affinity to HSA. In summary, our results demonstrate that it is possible to engineer two high affinity interactions into a small protein domain, hence rendering it bispecific without increasing the overall size, and the investigation also underlines the power of using different display technologies for different tasks in the engineering process.

## Materials and Methods

### Labeling of TNF-α

TNF-α (ProSpec-Tany TechnoGene Ltd, Rehovot, Israel) at a concentration of 0.15 mg ml^−1^ was biotinylated at room temperature (RT) using a 50-fold molar excess of EZ-Link™ Sulfo-NHS-LC-Biotin (Pierce, IL, USA) in phosphate buffered saline (PBS; 150 mM phosphate, 150 mM NaCl, pH 7.4). After 1 h incubation, an excess of glycine was added and the mixture was dialyzed against PBS over night (ON) at 4°C. The biotinylation was evaluated both by mass spectrometry and by immobilization on streptavidin-coated paramagnetic beads (Dynabeads® M-280, Streptavidin, Dynal A.S., Oslo, Norway) followed by sodium dodecyl sulphate polyacrylamide gel electrophoresis (SDS-PAGE).

### Phage display selection

TNF-α-binding molecules were selected by phage display from a combinatorial library based on an alkali-stabilized albumin-binding domain [17]. In the library (∼10^7^ variants), 11 amino acid positions have been diversified by an NNK-degeneracy resulting in 32 codons encoding all 20 amino acids and the amber stop codon (TAG) in each randomized position. In the first round of selection, 2.2·10^9^ *E. coli* cells (RRIΔM15 [21]) carrying the phagemid ABD-library were inoculated to 500 ml of tryptic soy broth supplemented with yeast extract (TSB+YE; Merck, Darmstadt, Germany), 2% glucose and 100 µg ml^−1^ ampicillin and grown to an OD_600 nm_ of 0.8. An aliquot of the cell culture (10 ml) was incubated with a 20-fold excess of helper phage (M13K07; New England Biolabs, MA, USA) for 30 min at 37°C. Infected cells were collected by centrifugation and used to inoculate 500 ml of fresh TSB+YE supplemented with 100 µg ml^−1^ ampicillin, 50 µg ml^−1^ kanamycin and 1 mM isopropyl β-D-thiogalactoside (IPTG; Apollo Scientific, Derbyshire, UK). Following ON cultivation, phages were isolated by two successive precipitation steps using ice-cold polyethylene glycol/sodium chloride (20% PEG6000/2.5 M NaCl). Phages were resuspended in PBS containing 0.1% Tween 20 and 3% BSA (TPBSB 3%) before being subjected to selection. For the last two rounds the volume of the ON culture was decreased to 100 ml.

Phages (5·10^12^ in the first round and a 1000-fold excess compared to the number of eluted phages in the following rounds) were transferred to a tube (Protein LoBind microcentrifuge tube; Eppendorf, Hamburg, Germany) blocked with PBS containing 0.1% Tween 20 and 5% BSA (TPBSB 5%) containing 0.2 mg neutravidin coated beads (REGEN beads, Dynal A.S.; coated with neutravidin according to supplier's recommendations) in 0.5 ml TPBSB 3%. After negative pre-selection for 30 min, the supernatant was transferred to a new tube containing 150 nM of biotinylated TNF-α (all stated concentrations of TNF-α refer to monomer, 17351 g mol^−1^) in a total volume of 1 ml TPBSB 3% followed by incubation for 2 h at RT. The mixture was transferred to a new tube containing washed and blocked neutravidin-coated paramagnetic beads, and incubated for 15 min to capture the complexes. The target concentration was decreased for each cycle (150–20 nM branched into four parallel tracks) whereas the number of washes and percentage of Tween 20 in the TPBSB 3% used for washing was increased. The same washing procedure as was used in the selection was also employed during pre-selection. For the last wash, a new blocked tube was used and the washing solution was changed to PBS. Bound phages were eluted by an addition of 500 µl 0.05 M glycine-HCl pH 2.2 and the eluate was neutralized with 50 µl 1 M Tris-HCl pH 8.0 and 450 µl PBS before it was used to infect a 200 ml log phase culture of RRIΔM15 *E. coli* for 30 min at 37°C. Cells were harvested by centrifugation and spread on tryptone yeast extract agar plates supplemented with 2% glucose and 100 µg ml^−1^ ampicillin. After ON incubation, the cells were collected and used to inoculate new TSB+YE medium, thereby starting the next round of selection.

### DNA-sequencing and subcloning

Phagemid inserts from individual clones from the third and fourth round of panning were PCR-amplified and sequenced with specific primers and Big Dye terminators (Amersham Biosciences, Uppsala, Sweden). Fragments were analyzed on an ABI Prism® 3700 DNA sequencer (Applied Biosystems, Foster City, CA). The same procedure was used to verify the sequences of DNA-fragments subcloned to the expression vector and cell-surface display vector.

Phagemids were purified from small-scale *E. coli* cultivations by EZNA plasmid purification kit (Omega Biotech, Victoria, Canada). ABD-inserts were PCR-amplified with primers introducing restriction sites for *Xho*I and *EcoR*I. The fragments were purified with MinElute PCR purification kit (Qiagen, Solna, Sweden) followed by restriction (*Xho*I and *EcoR*I; Fermentas). The expression vector, containing both a His_6_-tag and an inducible promoter, was prepared by plasmid preparation from RRIΔM15 *E. coli*, digested with the same enzymes and purified by preparative gel electrophoresis on a 1% agarose gel. After purification, the fragments were ligated with the vector using T4 DNA ligase (Fermentas) and transformed to RRIΔM15 *E. coli*. DNA sequences were verified followed by plasmid preparation and transformation to *E. coli* Rosetta (DE3) (Novagen, WI, USA) for protein production.

### Expression and purification of ABD-molecules

Single colonies were grown in 10 ml TSB medium containing 50 µg ml^−1^ kanamycin and 20 µg ml^−1^ chloramphenicol at 37°C ON. 100 ml of TSB+YE medium was inoculated with 1 ml of ON-culture and grown to log phase followed by induction of protein expression by addition of IPTG to a final concentration of 1 mM. The cultivations were incubated at 25°C ON and harvested by centrifugation (2700 g, 15 min, 4°C). The pellets were resuspended in 10 ml wash buffer (50 mM sodium phosphate, 6 M Urea, 300 mM NaCl, pH 7.5) and disrupted by sonication (Vibra Cell; Sonics and materials Inc., Danbury, CT, USA). Proteins were recovered from the supernatant after a subsequent centrifugation to remove cell debris (10000 g, 20 min, 4°C). The samples were filtered (0.45 µm) and loaded on a 1 ml Talon metal affinity resin column (Clontech Laboratories, CA, USA) equilibrated with eight column volumes (CV) of wash buffer. The column was washed with ten CV of wash buffer followed by elution in ten (1 ml) fractions (50 mM NaAc, 6 M Urea, 100 mM NaCl, 30 mM HAc, pH 4.5). Protein concentrations were estimated from absorbance measurements at 280 nm (Eppendorf Biophotometer) and the most concentrated fractions were analyzed by SDS-PAGE on Novex Bis-Tris 4–12% gradient gels using the Novex system (Invitrogen, CA, USA). Buffer exchange was done by extensive dialysis at 4°C against HEPES buffered saline (HBS-EP; 10 mM HEPES, 150 mM NaCl, 3.4 mM EDTA, 0.05% P20, pH 7.4). Concentrations were determined by amino acid analysis (Aminosyraanalyscentralen, Uppsala, Sweden) and molecular weights were verified by liquid chromatography electrospray ionization mass spectrometry (LC-ESI-MS) on a 6520 Accurate Mass Q-TOF LC/MS (Agilent Technologies, CA, USA).

### Biosensor analysis of first generation binders

Selected ABD-variants were screened for binding to HSA and TNF-α using surface plasmon resonance (SPR) with a BIAcore® 2000 instrument (Biacore, Uppsala, Sweden). HSA and TNF-α (both at 10 µg ml^−1^ in 10 mM NaAc, pH 4.5) were immobilized (2000 response units (RU) each) on a Biacore CM5 sensor chip by standard amine coupling. ABD-variants were serially injected over both flow cells at concentrations ranging from 1–5 µM with a flow rate of 50 µl min^−1^ at 25°C using HBS-EP as running buffer. The surfaces were regenerated between injections using 10 µl pulses of 10 mM HCl. The response from a reference flow cell was subtracted and the results were analyzed using BIAevaluation 3.2 software.

The two candidates that showed binding to TNF-α in the screening (ABD_TNF1_ and ABD_TNF2_) were immobilized, by amine coupling by the procedure described above, to 400 RU each on a new CM5 chip. TNF-α was injected (50 µl min^−1^ at 25°C using HBS-EP as running buffer) at concentrations ranging from 3.2–9960 nM. Injections were performed in duplicates and the kinetic constants (k_a_ and k_d_) were determined using BIAevaluation 3.2 software. To determine the affinity to HSA, both ABD-variants as well as the non-randomized ABD were injected in duplicates, at concentrations ranging from approximately 1–3000 nM (up to 250 nM for ABD), over a surface immobilized with HSA (2000 RU). Kinetic constants were determined as above and equilibrium responses (RU) from all experiments were fitted to a 1∶1 binding model using GraphPad Prism in order to determine the K_D_ values.

### Subcloning to staphylococcal display vector

The gene sequences encoding the non-randomized ABD and the two selected bispecific ABD-variants were PCR-amplified from their phage display vector constructs and ligated to a modified version of the previously described staphylococcal display vector pSCZ1 [22]. In the new vector (denoted pSCABD1), the albumin-binding protein (ABP) that was previously used for normalization of surface expression was replaced with a dimeric construct of an IgG-binding domain (Z_2_) derived from staphylococcal protein A. *E. coli* RR1ΔM15 was used as host for plasmid construction and preparation, and the constructs were transformed to electrocompetent *Staphylococcus carnosus* TM300 according to previously described protocol [23].

### Cell labeling and flow-cytometric analysis

Staphylococcal cells displaying ABD-variants were inoculated to 10 ml TSB+YE with 10 µg ml^−1^ chloramphenicol and grown ON at 37°C and 150 rpm. From ON cultures, approximately 10^6^ cells were washed with 1 ml PBS supplemented with 0.1% Pluronic® F108 NF Surfactant (PBSP; pH 7.4; BASF Corporation, Mount Olive, NJ). The cells were pelleted by centrifugation (3500 g, 4°C, 6 min) and resuspended in 100 µl of PBSP containing twelve different concentrations of biotinylated TNF-α (4.3–12900 nM) or fluorophore-conjugated HSA (1.25–2500 nM; 10–20000 nM for ABD_TNF1_). Equilibrium binding was reached by incubation at RT for 1 h with gentle mixing. The cells were washed with 1 ml ice-cold PBSP, followed by incubation on ice in 100 µl ice-cold PBSP containing 1.25 µg ml^−1^ streptavidin-Alexa Fluor 488 conjugate (Invitrogen) for 15 min (for HSA-binding the secondary incubation was omitted). Following one wash with 1 ml ice-cold PBSP, cells were resuspended in 300 µl ice-cold PBSP prior to flow-cytometric analysis. The mean fluorescence intensity (MFI) was measured using a FACS Vantage SE (BD Biosciences, San Jose, CA) flow cytometer. MFI data was fitted to a 1∶1 binding model using GraphPad Prism in order to determine the K_D_ values.

### Second-generation library construction and cloning

Two different degenerate oligonucleotides (Scandinavian Gene Synthesis AB, Köping, Sweden), encoding helix one and helix three, respectively, with complementary regions encoding the non-randomized second helix of ABD were annealed and extended by six cycles of PCR using AmpliTaqGold DNA polymerase (Applied Biosystems). Flanking restriction sites for *Xho*I and *Nhe*I were introduced in an additional PCR-reaction (15 cycles) using a new primer pair. The PCR-products were purified with QIAquick PCR Purification Kit (Qiagen) according to the supplier's recommendations. The purified pool of randomized library fragment was digested with restriction enzymes *Xho*I and *Nhe*I (New England Biolabs) and purified as above. The staphylococcal display vector, pSCABD1, was prepared from *E. coli* RR1ΔM15 and purified using Jetstar Maxi Kit (Genomed, Bad Oeynhausen, Germany). The vector was digested with the same enzymes and purified using preparative gel electrophoresis on a 1% agarose gel. Ligation of pSCABD1 with the randomized library fragments was performed at a 1∶20 molar ratio of vector to fragment using T4 DNA ligase (New England Biolabs). The ligation mixture was purified using QIAquick Gel Extraction Kit according to the supplier's recommendations prior to transformation to electrocompetent *E. coli* DH5α cells. Individual clones, plated directly after transformation, were PCR-amplified for sequence verification using BigDye Thermo Cycle Sequencing reactions and an ABI Prism® 3700 instrument (Applied Biosystems). Plasmids were prepared from ON cultures of *E. coli* using Jetstar Maxi Kit (Genomed) and transformed to electrocompetent *S. carnosus* as described above. The staphylococcal library is hereinafter denoted Sc∶ABD_TNFlib_.

### Cell labeling and FACS

Cells, approximately 50 times the library size, were labeled basically as described above, but with the addition of fluorescently conjugated IgG in the second incubation for monitoring of the surface expression level and normalized gating. The labeled cell-displayed library was sorted using a FACS Vantage SE (BD Biosciences) flow cytometer. The sort gate was set to sort out the top fraction of ABD-displaying cells showing the highest Alexa Fluor 488 (TNF-α-binding through labeled streptavidin) to Alexa Fluor 647 (IgG-binding) fluorescence intensity ratio. 1 µM biotinylated TNF-α was used in the first round and concentrations down to 1 nM in following rounds. Cells were sorted directly into 5×1 ml TSB+YE and thereafter inoculated to TSB+YE containing 10 µg ml^−1^ chloramphenicol and incubated at 37°C for approximately 38 hours in order to amplify isolated cells by growth for the next round of labeling and FACS. The procedure was repeated three times using different concentrations of biotinylated TNF-α and HSA conjugated to Alexa Fluor 488. In experiments where the cell library was labeled with both TNF-α and HSA, streptavidin conjugated to R-phycoerythrin was used for detection of biotinylated TNF-α and HSA-Alexa Fluor 488 conjugate was added in the second incubation. After the third sorting round, isolated cells were spread on agar plates and selected variants were identified by DNA-sequencing.

### Subcloning, expression, purification and characterization of second-generation ABD-molecules

Six chosen candidate binders (ABD_T001_, ABD_T002_, ABD_T004_, ABD_HT014_, ABD_HT015_ and ABD_HT016_) from the two selection strategies were subcloned from the staphylococcal cell surface display vector to the expression vector using specific oligonucleotide primers essentially as described above. The resulting constructs were sequence verified and plasmids transformed into *E. coli* Rosetta (DE3). Protein expression was carried out as described above but purification was based on albumin binding by HSA affinity chromatography. Pelleted bacteria were resuspended in 10 ml tris-buffered saline (TST; 25 mM Tris-HCl, 200 mM NaCl, 1 mM EDTA, 0.5% (w/v) Tween 20, pH 8.0) and disrupted by sonication. The lysates were loaded on 4 ml HSA-sepharose (Pharmacia Biotech) gravity flow columns equilibrated with TST. The columns were washed with 20 CV TST followed by 12 CV of 5 mM NH_4_Ac pH 5.5 and eluted by 0.5 M HAc in 1 ml fractions. Eluted fractions were dried by speedvac (Savant AES2010, Thermo Scientific, MA, USA) over night, dissolved in PBS and the purity was evaluated by SDS-PAGE. Concentrations were determined by amino acid analysis (Aminosyraanalyscentralen, Uppsala, Sweden) and molecular weights were verified by LC-ESI-MS.

### Biosensor analysis of second-generation binders

The binding kinetics of the six selected second-generation molecules for the two targets were determined by SPR on a ProteOn XPR36 Protein Interaction Array System (Bio-Rad, CA, USA). The ABD-molecules were diluted to 10 µg ml^−1^ in 10 mM NaAc pH 4.5 and immobilized by amine coupling on a ProteOn GLM sensor chip (approximately 800–1500 RU) followed by injection of dilution series of HSA or TNF-α (50 µl min^−1^ at 25°C using PBS supplemented with 0.1% Tween 20 as running buffer). The analysis was repeated at least in triplicates for each interaction, on two different sensor chips (GLC in the second run) and two different days using different dilution series of the analytes (ranging from 1–100 nM for TNF-α and 7–1200 nM for HSA).

In addition, simultaneous binding of the bispecific ABD-molecules to HSA and TNF-α was investigated on a chip with HSA immobilized in two flow cells to different surface densities. In a first experiment 100 nM of the bispecific ABD-molecules ABD_T001_ or ABD_HT014_ were injected over the HSA-surfaces followed by injection of a dilution series of TNF-α (starting at 100 nM). Furthermore, 100 nM ABD_T001_ or ABD_HT014_ were injected over the two surfaces and compared to 100 nM of the same molecules pre-incubated for 10 min with a 20-fold molar excess of TNF-α. A competition assay with a TNF-α-binding affibody molecule (Z_185_; [24]) based on a closely related protein scaffold was set up with one of the best performing TNF-α-binding molecules (ABD_T001_). Both molecules were immobilized in separate channels on a sensor chip and 100 nM TNF-α or 100 nM TNF-α pre-incubated for 10 min with a 20-fold molar excess of ABD_T001_ or Z_185_ was injected. The responses from the pre-incubated samples were compared to TNF-α injected without competitor.

## Results

### Phage display selection

**Figure 1:**
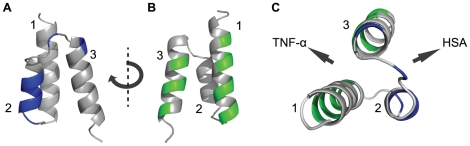
Structure of the three-helical albumin-binding domain (ABD) from streptococcal protein G (PDB: 1GJT). Helices are numbered as 1, 2 and 3 and indicated in the figure. **A**. Indicated in blue are amino acids on helix 2 and 3 (S18, Y20, Y21, K22, N23, L24, K29 and E32) that have been suggested to participate in the interaction to albumin (defined as resulting in at least a 2-fold decrease in affinity when mutated to alanine [16]). **B**. Indicated in green are amino acids on helix 1 and 3 that were targeted for randomization in the library. The library was designed in order to randomize a surface that was non-overlapping with the positions that are interacting with albumin. **C**. Structure of ABD observed from above with the albumin-interacting surface on helix 2 and 3 indicated in blue and the randomized surface on helix 1 and 3 indicated in green.

**Figure 2:**
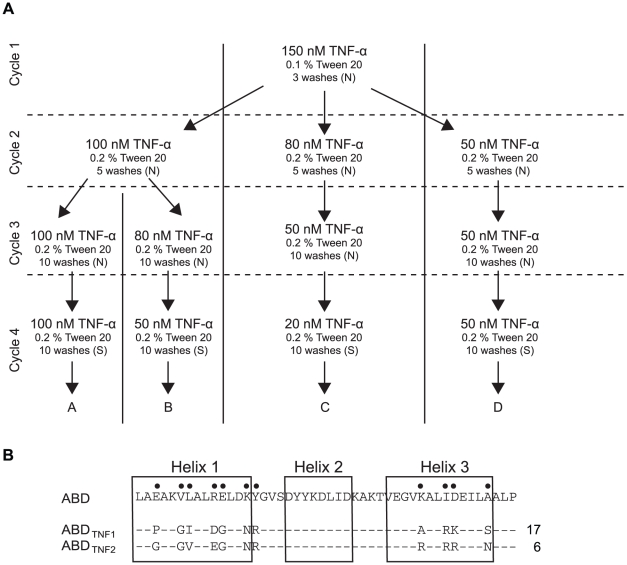
Selection strategy for phage display enrichment of TNF-α-binding ABD-variants and sequences of bispecific first-generation molecules. A. Four cycles of selection, split into four parallel tracks, were performed. The percentage of Tween 20 and the number of washes were increased in each round of selection. Neutravidin coated paramagnetic beads (N) were used to capture phage-target complexes in cycles 1-3, and in cycle 4 streptavidin coated beads (S) were used. B. Amino acid sequences of the bispecific variants ABD_TNF1_ and ABD_TNF2_ and the number of times those clones were observed. Non-randomized ABD is shown as a reference and randomized positions are indicated with bullets.

In order to generate new potentially bispecific affinity proteins, a phage-displayed combinatorial library was used in biopannings with TNF-α as target. The library is based on an alkali-stabilized form of an albumin-binding domain (ABD) from streptococcal protein G. Eleven positions on helix one and helix three that are not involved in the inherent HSA interaction are randomized with NNK degenerated codons (Fig. 1) [17]. Four rounds of biopanning (A–D) against TNF-α were performed for isolation of binders, with decreasing target concentration and an increasing number of washes in each successive cycle (Fig. 2A). Sequencing of clones from the third and the fourth round of panning revealed 19 unique candidates, out of which six occurred more than once and were therefore selected for further characterization. Moreover, the two clones that occurred most frequently (denoted ABD_TNF1_ and ABD_TNF2_) showed a high degree of sequence similarity (Fig. 2B) and were also found in several parallel selection tracks, reflecting independent enrichment.

### Biosensor analyses of selected ABD-variants

To produce soluble ABD-variants for binding studies, the six selected candidates were subcloned into a production vector containing an N-terminal His_6_-tag. The corresponding proteins were expressed in *E. coli* and purified to homogeneity. Correct molecular weights were verified by LC-ESI-MS (data not shown). The six ABD-variants were thereafter screened for binding to TNF-α and HSA in an SPR-based biosensor assay, demonstrating retained binding to HSA, although several had lower affinities compared to the non-randomized ABD-molecule (data not shown). Furthermore, the two most frequently observed candidates in the sequencing (ABD_TNF1_ and ABD_TNF2_) were also binding to immobilized TNF-α (Table 1), indicating that the biopanning had been successful. The two candidates demonstrating dual binding were immobilized on a new sensor chip and subjected to binding analyses to determine their affinities to the two target proteins. Apparent equilibrium dissociation constants for both target molecules were obtained by non-linear regression of the SPR data. ABD_TNF1_ showed a K_D_ of approximately 400 nM for TNF-α and 1900 nM for HSA (Table 1). Interestingly, ABD_TNF2_ had instead a high affinity for HSA (17 nM) and a moderate apparent affinity for TNF-α (1600 nM) (Table 1). Functional binding to albumin was also verified for ABD_TNF1_ and ABD_TNF2_ in affinity-chromatography purification, using immobilized HSA as ligand on the matrix [25]. The purification was successful with no detectable recombinant protein in the flow through and pure proteins of correct size in the eluate (data not shown). In addition to verifying the albumin-binding capacity of the two new variants, the results also demonstrate that bispecific ABD-molecules can be produced and efficiently purified without an additional purification tag, which further reduces the size of the final affinity protein. This production and purification strategy should be applicable to all future bispecific ABD-based binders, regardless of their engineered additional specificity.

**Table 1:** Affinities (K_D_) and kinetic parameters (k_a_ and k_d_) for ABD_TNF1_, ABD_TNF2_ and non-randomized ABD.

	SPR						On-cell	
	HSA			TNF-α			HSA	TNF-α
	k_a_	k_d_	K_D_	k_a_	k_d_	K_D (app)_	K_D_	K_D (app)_
	[M^-1^s^-1^]	[s^-1^]	[nM]	[M^-1^s^-1^]	[s^-1^]	[nM]	[nM]	[nM]
ABD_TNF1_	9.1·10^3^	0.02	1850	7.8·10^4^	0.03	385	>2000(*)	267
ABD_TNF2_	6.0·10^4^	1.0·10^-3^	17	2.3·10^4^	0.04	1560	23	594
ABD	1.7·10^5^	9.0·10^-4^	5.3	-	-	-	19	-

(*) Saturation was not reached for the highest concentration (20 µM).

Binding to HSA and TNF-α was determined by surface plasmon resonance spectroscopy (SPR) or on-cell by flow cytometry. Kinetic constants from SPR-measurements were measured on duplicate samples and K_D_ calculated from the mean values. On-cell measurements were performed once for HSA and in duplicates for TNF-α. All parameters for TNF-α are calculated from the concentration of monomeric protein.

### On-cell evaluation and construction of a cell-displayed affinity maturation library

**Figure 3:**
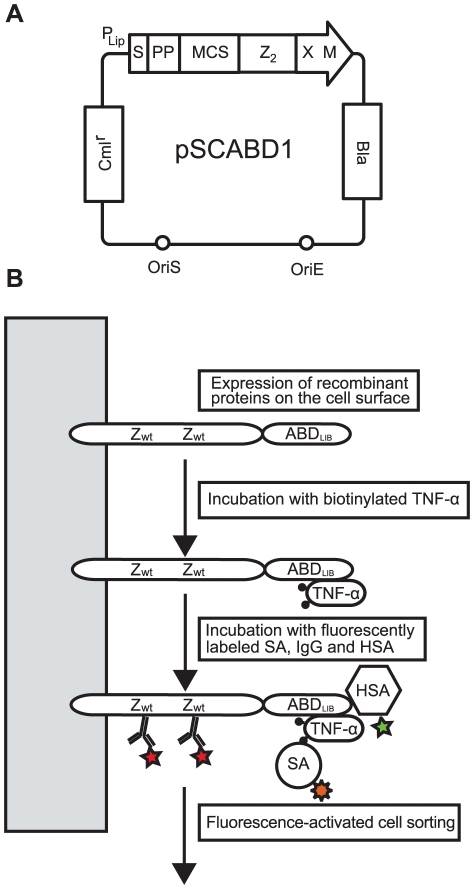
Staphylococcal display vector and schematic representation of the cell-surface displayed ABD-library. **A**. Staphylococcal display vector for display of heterologous proteins on the surface of *S. carnosus*. Abbreviations: Z2, dimeric Z-domain gene; Bla, beta-lactamase encoding gene; Cmlr, chloramphenicol acetyl transferase-encoding gene; OriE and OriS, origin of replication for *E. coli* and staphylococci, respectively; PP, propeptide from *S. hyicus* lipase; PLip, promoter from *S. hyicus* lipase. S, signal sequence from *S. hyicus* lipase; XM, cell wall anchoring region from staphylococcal protein A. **B**. Schematic representation of the recombinant ABD-library in fusion to the dimeric Z-domain displayed on the staphylococcal cell surface. The dimeric Z-domain functions as a reporter tag for monitoring of the surface expression level of individual cells through binding to labeled IgG. The target-binding signal can thereby be normalized with the expression level in order to minimize expression bias during sorting. Labeled proteins in the first and secondary incubations for the dual selection are indicated in the figure.

To further characterize the two most promising anti-TNF-α candidates from the phage display selection and to verify functional expression on bacterial cells, staphylococcal surface display was employed for an on-cell affinity measurement using flow cytometry. Monomeric constructs of the two variants were subcloned into a staphylococcal display vector for subsequent transformation to the staphylococcal host and expression on the cell surface (Fig. 3A and B). Staphylococcal cells displaying the ABD-molecules in fusion with a dimeric Z-domain, were incubated with different concentrations of biotinylated TNF-α (4.3 to 12900 nM) or HSA-Alexa 488 conjugate (1.25–2500 nM, concentrations up to 20 µM for ABD_TNF1_). As a reference, non-randomized ABD was also subcloned, expressed and incubated with the same dilutions of fluorophore-labeled HSA and TNF-α. After washing and incubation with fluorescently labeled streptavidin (for detection of TNF-α) cells were analyzed by flow cytometry. The results showed a high surface expression level for all three recombinant proteins as well as specific binding to TNF-α and HSA for ABD_TNF1_ and ABD_TNF2_, while the non-randomized ABD was only binding HSA and not TNF-α. Mean fluorescence intensities (MFI) from the flow-cytometric analysis were fitted using non-linear regression to a monovalent binding equation for determination of K_D_ values and the affinities were in concordance with the affinities determined by SPR (Table 1).

**Figure 4:**
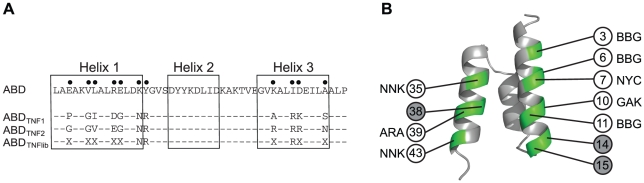
Randomized positions in the affinity maturation library (Sc∶ABD_TNFlib_). **A**. Sequence in randomized positions of the first generation binders ABD_TNF1_, ABD_TNF2_ and Sc∶ABD_TNFlib_. **B**. Positions targeted for randomization indicated by light circles. Three positions randomized in the initial library were omitted in the design, indicated by dark circles. The randomizations are listed beside the position. ARA  =  Arg, Lys. BBG  =  Ala, Arg, Gly, Leu, Pro, Ser, Trp, Val. GAK  =  Asp, Glu. NYC  =  Ala, Ile, Leu, Phe, Pro, Ser, Thr, Val. NNK  =  all amino acids and one amber stop. The theoretical size of the library is 6.6·10^6^.

Although the phage display selection was successful and yielded two variants that demonstrated binding to both HSA and TNF-α, the affinities were moderate and the candidate that was displaying the highest apparent affinity for TNF-α had around 400-fold lower affinity for HSA compared to the non-randomized ABD. To further improve the ABD-variants, an affinity maturation library was designed with the aim to primarily increase the affinity for TNF-α (Fig. 4). However, the more challenging goal to engineer high affinity to both TNF-α and HSA into the same binder was also envisioned. The design was based on the two most prevalent first generation binders that showed affinity to both HSA and TNF-α. Three of the positions that were identical in ABD_TNF1_ and ABD_TNF2_ were locked and the remaining eight of the previously selected eleven positions were targeted for randomization, although with less diversity in most positions (Fig. 4). This diversification resulted in a library with a theoretical complexity of approximately 6·10^6^ variants. Since cell display provides the ability to analyze the library for TNF-α- and HSA-binding simultaneously in a flow cytometer, the Gram-positive bacterium *Staphylococcus carnosus* was chosen as display host for the affinity maturation [26]. In addition, cell display has proven particularly suitable for affinity maturation efforts, mainly due to an excellent affinity discrimination capacity during FACS [26], [27], [28]. The library was transformed to staphylococci, yielding roughly 2·10^7^ transformants, hence covering the theoretical complexity almost 3-fold. 360 clones from the unsorted library were sequenced and showed a good agreement compared with the design (data not shown). Full-length ABD-sequences were found in 94% of the sequenced clones, 4% contained an amber stop codon in a randomized (NNK) position and less than 2% showed deletions, duplications or mutations that were not in agreement with the design. To determine the proportion of variants that had retained a detectable HSA-binding after randomization, the library was incubated with fluorescently labeled IgG as well as HSA and thereafter analyzed in the flow cytometer. The analysis showed that around 50% of the population was still able to bind to HSA, suggesting that the library design was successful and indeed targeted mainly to the part of ABD that is not involved in the interaction with HSA as intended.

### Flow-cytometric sorting for isolation of improved ABD-molecules

**Figure 5:**
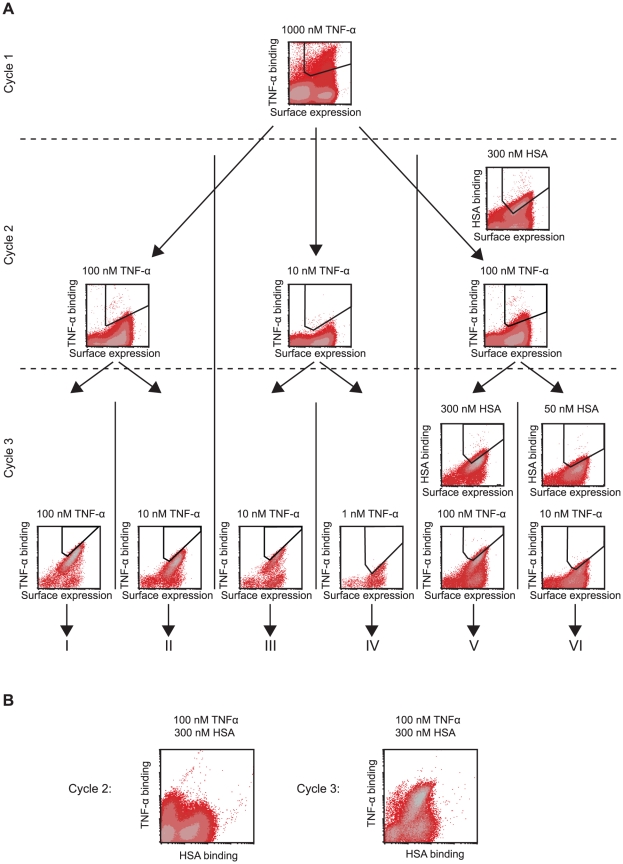
Density plots showing the results from flow-cytometric sortings of Sc∶ABD_TNFlib_. **A**. The density plots are showing the staphylococcal library before flow-cytometric sorting round 1, 2 and 3, respectively, with regions used for gating outlined in each plot. The target protein and the concentration used in each sorting are indicated on top of respective density plot. FL-4 channel fluorescence intensity corresponding to surface expression level (monitored via IgG-binding) on the x-axis and FL-1 channel fluorescence corresponding to TNF-α- or HSA-binding on the y-axis. **B**. Density plots showing the analysis of bispecificity of Sc∶ABD_TNFlib_ in cycle 2 and cycle 3 (incubated with 100 nM TNF-α and 300 nM HSA). FL-1 channel fluorescence intensity corresponding to HSA-binding on the x-axis and FL-2 channel fluorescence corresponding to TNF-α-binding on the y-axis.

For isolation of variants with improved affinity for TNF-α and HSA respectively, the staphylococcal library was subjected to three rounds of FACS with alternating rounds of amplification by cell growth. In total, six sorting strategies with different TNF-α concentrations were employed (I-VI, Fig. 5A). For two of the six strategies (V-VI), the library was simultaneously incubated with both TNF-α and HSA (labeled with different fluorophores) from round two in order to actively sort for bispecific variants (Fig. 3B). In all selection rounds, fluorescently labeled IgG was used for surface expression normalization through binding to the dimeric Z-domain. Selection stringency in terms of target concentration and sorting parameters was increased with each sorting round and typically the top 0.5–1% of the library, that demonstrated the highest target-binding to surface expression ratio, was gated and isolated for amplification and subsequent rounds of sorting (Fig. 5A). One of the advantages with cell-based selection systems is the straightforward monitoring of the obtained enrichment of target-binding cells in the flow cytometer. In this study, the visualization of the target-binding properties of the library revealed an enrichment of target-binding clones in each sorting round (Fig. 5A). For the dual selections (V and VI), TNF-α and HSA-binding was also monitored in an additional dot plot, visualizing potential bispecificity of individual cells (Fig. 5B). The bispecificity analysis revealed that after the first cycle, a majority of the library was mostly monospecific, i.e. demonstrating a detectable affinity for either TNF-α or HSA. However, after the dual selection in cycle two, the bispecific population was significantly enriched, thus verifying that efficient enrichment of two distinct specificities may be obtained in one single sorting using our strategy (Fig. 5B). It should be noted though that detection of fluorescent signals corresponding to binding of both HSA and TNF-α is only demonstrating simultaneous binding on the cell level but not on a molecular level. This is due to the multivalent display of recombinant proteins on the cell surface where a part of the protein population might bind to HSA and another part to TNF-α, resulting in signals in both fluorescence channels. Such clones are indeed bispecific, but each single ABD-molecule is not necessarily capable of simultaneous binding. In addition, after the first cycle, a control experiment was performed in which the library was incubated with only secondary reagent (streptavidin-Alexa Fluor 488) to confirm that isolated clones were specific for TNF-α and not streptavidin. The results showed no binding and thereby verified that the enriched specificity was for the target proteins. After three rounds of FACS, isolated cells were spread on agar plates for sequencing and characterization of individual candidates.

**Figure 6:**
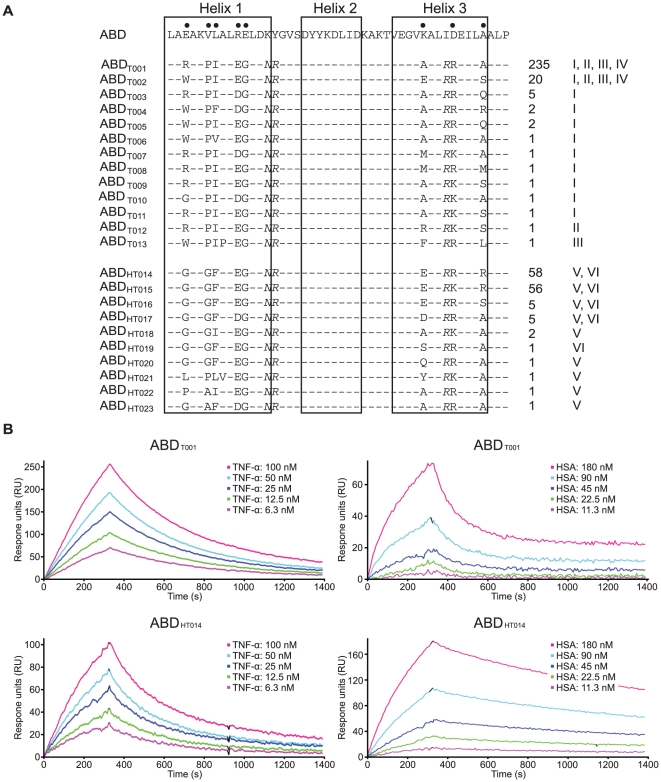
Amino acid sequences, origin and binding affinities of clones from the third round of FACS. **A**. The sequence of non-randomized ABD is shown on top with the eight positions subjected to randomization in Sc∶ABD_TNFlib_ indicated. Boxes denote the localization of the three helices in the structure of the parental ABD-molecule. The candidates are named and grouped according to selection strategy. Variants from selections targeting only TNF-α (tracks I-IV, ABD_T001-013_) and from the dual-selection (tracks V-VI, ABD_HT014-023_) are shown. The columns to the right indicate the number of times each clone was identified by DNA-sequencing and the selection track(s) from where the sequence originated. **B**. Representative sensorgrams from SPR-analysis of immobilized ABD_T001_ and ABD_HT014_ binding to TNF-α and HSA. TNF-α was injected at concentrations ranging from around 6–100 nM and HSA from 10–180 nM, data are double referenced by subtraction of simultaneous responses from interspots and a buffer injection.

By sequencing in total 403 colonies from all of the six sorting strategies, 23 unique ABD-variants were identified. The most prevalent occurred 235 times and the least prevalent clones were found only once (Fig. 6A). Interestingly, no overlap was seen between sequences from the bispecific sortings (V and VI) and sequences from the TNF-α sortings (I-IV), i.e. no clones identified from sortings V and VI were found among the clones from sortings I-IV and vice versa (Fig. 6A). In addition, the homology between these two sets of sequences was also lower compared to the homology within each set. For sorting strategies employing a more stringent selection pressure with lower target concentration, the number of different clones was lower, indicating a successful and converging enrichment of high-affinity variants (Fig. 6A). Two sequences with mutations in scaffold positions were observed (ABD_T013_ and ABD_HT021_).

### On-cell evaluation of second-generation binders

All the 23 clones were screened for binding to both TNF-α and HSA at two concentrations each (10 and 150 nM). The analysis was performed on the cell surface and the target-binding of each variant was measured using flow cytometry. The analysis showed that all selected clones bound their respective targets in a concentration-dependent manner (data not shown). Interestingly, all clones derived from the dual selection demonstrated a higher relative affinity for HSA compared to the clones selected using only TNF-α. However, variants with high TNF-α-binding signals were found in all tracks, suggesting that the selection pressure for bispecificity is affecting the selection but not limiting the affinity to a very large extent. In summary, all 23 different isolated variants were able to bind to both TNF-α and albumin in the flow cytometer, demonstrating that the flow-cytometric sorting was successful. Based on these results, the three best performing clones from each strategy (ABD_T001_, ABD_T002_, ABD_T004_, ABD_HT014_, ABD_HT015_ and ABD_HT016_) were selected for further on-cell binding analyses using flow cytometry as well as expression and subsequent HSA-affinity purification.

Binding to albumin has been shown to extend the *in vivo* half-life of proteins, and is considered to have great potential as a strategy for increasing the potency of biological drugs based on small affinity proteins. This mechanism, which depends on binding to the neonatal Fc-receptor (FcRn) at acidic pH in the endosomes, has been demonstrated for antibodies, albumin as well as for various albumin-binding moieties [18], [19]. In order to get a first indication whether our new bispecific binders would be suitable for *in vivo* applications, two experiments were performed, assessing both the important pH-dependency as well as evaluating if the ABD-variants could bind TNF-α in an excess of albumin, thus mimicking the conditions in blood. In the first experiment, the six selected binders were evaluated on the cell surface to assess the pH-dependency of the HSA-interaction by measuring the off rate from albumin at both pH 7.4 and 5.5. No significant difference in dissociation rate from HSA could be detected for the different bispecific variants when binding at pH 7.4 was compared to pH 5.5 (data not shown), and the results thus support the hypothesis that the bispecific molecules may be able to utilize FcRn-mediated recycling for half-life extension. In addition, the TNF-α-binding ability in presence of an excess of HSA was evaluated to mimic the natural conditions *in vivo*. Cells expressing the bispecific ABD-variants were first pre-incubated with saturating concentrations of HSA before being incubated with TNF-α in presence of a large excess of HSA. The signal from the fluorescently labeled TNF-α was measured by flow cytometry and the results showed that the TNF-α-binding on-cell could be detected for all six variants, reaching almost the same levels as without pre-incubation with HSA (data not shown). Moreover, the TNF-α-binding signal in presence of a large excess of HSA was higher for the variants with a lower HSA affinity compared to variants from the dual selection that exhibit a higher HSA affinity. The results hence indicate that the binding of TNF-α interferes with albumin binding, but that the stronger binding to TNF-α can compete with the interaction to HSA. As expected, the TNF-α-binding capacity in presence of a large excess of HSA was higher for the variants that were only selected against TNF-α as compared to candidates from the dual selection.

### Affinity determination of bispecific candidates

In order to further investigate the ability of the domains to bind the two different target proteins, the six selected second-generation candidates were expressed in *E. coli* and affinity purified based on their HSA-binding ability. The results demonstrated again that the bispecific ABD-molecules could be expressed and efficiently purified as single domains without an extra purification tag.

The soluble ABD-variants were immobilized on sensor chip surfaces for determination of the affinities to TNF-α and HSA using SPR (Fig. 6B). Kinetic evaluation of the binding of the six ABD-variants to TNF-α revealed apparent K_D_ values in the low nanomolar range (down to 2.9 nM), which corresponds to a more than 100-fold improvement in affinity compared to the best first-generation binder (Table 1 and 2). In addition, the candidates from the dual selection (ABD_HT014_, ABD_HT015_ and ABD_HT016_) showed a high affinity to both targets, with around 5 nM apparent affinities for TNF-α and 35–70 nM for HSA (Table 2). The results thus demonstrate that using the dual selection strategy, the relatively moderate affinity of the first-generation clones to TNF-α and HSA, respectively, could be increased but also combined into potent bispecific affinity proteins.

**Table 2:** Affinities (K_D_) and kinetic parameters (k_a_ and k_d_) for six selected affinity matured binding molecules.

	HSA			TNF-α		
	k_a_	k_d_	K_D_	k_a_	k_d_	K_D (app)_
	[M^-1^s^-1^]	[s^-1^]	[nM]	[M^-1^s^-1^]	[s^-1^]	[nM]
ABD_T001_	6.4 (±3.3) 10^3^	1.2 (±0.1) 10^-3^	192	3.7 (±1.2) 10^5^	1.9 (±0.3) 10^-3^	5.2
ABD_T002_	2.4 (±1.2) 10^3^	1.6 (±0.08) 10^-3^	649	5.0 (±3.1) 10^5^	1.5 (±0.5) 10^-3^	2.9
ABD_T004_	3.9 (±1.1) 10^3^	8.1 (±0.7) 10^-4^	209	5.3 (±2.6) 10^5^	2.1 (±0.4) 10^-3^	4.0
ABD_HT014_	1.4 (±0.6) 10^4^	5.4 (±0.3) 10^-4^	39	5.8 (±3.1) 10^5^	2.9 (±1.1) 10^-3^	5.0
ABD_HT015_	1.3 (±2.0) 10^4^	4.6 (±0.2) 10^-4^	35	4.5 (±2.5) 10^5^	2.0 (±0.3) 10^-3^	4.4
ABD_HT016_	8.9 (±1.4) 10^3^	6.1 (±0.2) 10^-4^	68	4.2 (±2.0) 10^5^	1.9 (±0.5) 10^-3^	4.5

ABD_T001_, ABD_T002_, ABD_T004_, ABD_HT014_, ABD_HT015_ and ABD_HT016_ binding to HSA and TNF-α. Kinetic constants from SPR-measurements were determined in triplicates on two different sensor chips on two different days and K_D_ was calculated from the mean values of the rate constants. Standard deviations are shown and all parameters for TNF-α are calculated from the concentration of monomeric protein.

### Binding assays

Although several of the isolated binders from the affinity maturation had a high affinity to both TNF-α and albumin, it was not possible to determine if they were able to bind the two targets simultaneously from the on-cell experiments. In order to investigate whether our binders were capable of simultaneous binding, we performed two different biosensor assays. In the first experiment, the bispecific proteins were injected over a surface immobilized with HSA and followed by co-injections of TNF-α. In the second experiment, the bispecific proteins were instead pre-incubated with TNF-α and thereafter injected over a surface immobilized with HSA. No simultaneous binding could be detected in any of the two assay formats. The results hence indicate that the findings from the flow-cytometric assay, where TNF-α-binding was detected in presence of an excess of HSA, was indeed due to the much higher affinity for TNF-α compared to the affinity for HSA (10 to 100-fold) and not due to simultaneous binding. Even though the new bispecific binders demonstrated a high affinity to both TNF-α and albumin, the results were not surprising. The ABD-variants are comprised of only 46 amino acids and both targets are relatively large, probably resulting in sterical hindrance and thus no opportunity for simultaneous binding. However, it is too early to draw any general conclusions from this result and selections against other targets must be performed to definitely assess the possibility of simultaneous binding.

As mentioned above, ABD is a small domain with a three-helical fold. Although it is not related in primary sequence, it demonstrates a high structural homology to the well-investigated affibody molecules [11], which are derived from protein A. TNF-α specific affibody molecules have been generated in a previous study and due to their similar structure we wanted to investigate if the two different classes of binders were directed against the same epitope on TNF-α. Consequently, an SPR-based competition assay was performed with the most prevalent bispecific protein (ABD_T001_) and a well-characterized affibody molecule specific for TNF-α [24]. The analysis showed that pre-incubation of TNF-α with either of the binders efficiently blocked the interaction to the other binder that was immobilized on a sensor chip surface (data not shown). The results hence suggest that our new bispecific binders are directed against the same epitope as the affibody molecule, which is interesting since both affinity proteins are based on small three-helix bundle scaffolds where the engineered binding surface is accommodated on a rather flat surface.

## Discussion

Small size and a long *in vivo* half-life are two important properties for affinity proteins in many therapeutic applications. Unfortunately, fulfilling both requirements is generally difficult, as small size of the protein tends to result in a rapid excretion from the body. However, the *in vivo* half-life of molecules can be increased considerably by binding to human serum albumin (HSA). Hence, a small bispecific molecule with capability to bind a specific target protein and also HSA would potentially combine the favorable features linked to small size with a prolonged half-life *in vivo*. In order to generate molecules with those characteristics, an alkali-stabilized variant of one of the albumin-binding domains (ABD) from streptococcal protein G was used as starting molecule for the construction of a combinatorial protein library. Guided by the previously solved structure of a closely related albumin-binding domain interacting with HSA [15] and by previously reported alanine-scanning data of the ABD/HSA interaction [16], eleven positions were targeted for randomization [17]. The selected positions were distributed on helix one and helix three, i.e. on the opposite side to the albumin-binding region, in order to increase the probability of obtaining bispecific proteins (Fig. 1). The resulting library was displayed on phage particles and subjected to biopanning against the disease-related tumor necrosis factor-α (TNF-α). Sequencing of isolated variants revealed two dominating clones that were further characterized. Not surprisingly, the apparent affinity for TNF-α was relatively moderate with a K_D_ of around 400 nM for the stronger variant and 1600 nM for the weaker (Table 1). However, the binding capacity to HSA was still detectable, although considerably decreased for one of the binders. Interestingly, the variant exhibiting the strongest affinity for TNF-α (ABD_TNF1_) showed a pronounced decrease in affinity for HSA, which illustrates the challenge in engineering two high affinity interactions into one small domain. The interactions were also analyzed by flow cytometry with the binding molecules displayed on staphylococcal cells, demonstrating affinity for both TNF-α and HSA, and at the same time establishing the utility of this platform for further improvements (Table 1, Fig. 3). In order to primarily increase the apparent affinity for TNF-α and potentially combine high affinity for both TNF-α and HSA in one binder, an affinity maturation library was designed, subcloned and expressed on the surface of staphylococci. The choice of cell display over phage display for the affinity maturation was based on several potential advantages with the former. First, cell display in combination with quantitative FACS is particularly powerful for affinity maturation purposes, where improved second-generation variants must be discriminated from a background of binders with moderate affinity [26], [27], [28]. Moreover, the possibility of monitoring several parameters simultaneously in the flow cytometer enables sorting for bispecific binders that show binding to both targets in the same sorting cycle. The library design was based on the two bispecific candidates isolated by the phage display selection, resulting in a theoretical complexity of almost seven million variants (Fig. 4). Prior to FACS, the cell library was incubated with TNF-α and IgG labeled with two different fluorophores. This provided means to monitor the surface expression level of individual cells and thereby perform normalization for fine affinity discrimination. In later selection cycles, the labeling complexity was further increased by employing a dual selection strategy in order to simultaneously detect: i) bound TNF-α, ii) bound HSA and iii) surface expression level (bound IgG) of every individual cell, which the sorting was based on. Enrichment of target-binding cells was demonstrated already in the flow cytometer after respective sorting cycle. After three rounds of FACS, genes of displayed proteins of individual cells were sequenced for identification of improved candidates (Fig. 5A). Interestingly, in cycle two, the three-color labeling revealed that the majority of the clones in the library were not able to bind to both TNF-α and HSA, showing only binding to either one of them. However, after applying a two-gate sorting to isolate cells with affinity for both TNF-α and HSA (strategies V and VI), the double-positive population was significantly enriched in subsequent rounds (Fig. 5B). Moreover, sequencing of these cells revealed a unique set of candidates with relatively low homology compared to the set isolated solely for TNF-α-binding (Fig. 6A). On the other hand, the bispecific binders showed a high similarity to the first-generation binder exhibiting the strongest HSA affinity (ABD_TNF2_). The TNF-α and HSA affinities of the clones showing highest signals in a first screening experiment were determined by surface plasmon resonance spectroscopy. In terms of TNF-α-binding, the analysis revealed low nanomolar affinities corresponding to around a 100-fold improvement for the best performing candidate compared to the best first-generation clone (Table 2). Interestingly, strong TNF-α binders were isolated from both the selection using only TNF-α as target and the dual strategy, suggesting that retaining the high affinity for HSA does not necessarily limit the affinity for TNF-α. Bispecific binders with affinities below 40 nM for HSA and around 5 nM for TNF-α were isolated, demonstrating the power of the new dual sorting strategy.

Although similar results may have been achieved using phage display and alternating rounds of selection to respective target, the possibility of using multiparameter sorting and monitoring of the library during selection makes cell display particularly well suited for these efforts. Even though the binding to HSA was measurable for a large part of the variants in the maturation library, the randomization and selection resulted in rather low affinity for HSA for some clones, especially when performing the selection only against TNF-α (Table 1 and 2). However, in a recently published study, Nygren and coworkers were able to improve the binding between ABD and HSA almost 1,000,000-fold, i.e. down to femtomolar affinity [29]. Interestingly, one of the beneficial mutations (K35E) discovered in that paper was also enriched after the dual selection, indicating that simultaneous improvement of both interaction surfaces is possible. If necessary, grafting more of these mutations into the candidates generated here might further improve the affinity for HSA for our clones as well. On the other hand, recent studies have demonstrated that even a weak or moderate affinity for albumin is sufficient to achieve half-life extension [30]. Interestingly, the six selected clones all demonstrate ability to bind TNF-α in the presence of high concentrations of HSA, despite the inability to bind both targets simultaneously. This is due to the much higher affinity towards TNF-α than HSA for all our binders. However, which affinities that will be optimal *in vivo* and if simultaneous binding is a prerequisite, is something that must be investigated further using appropriate animal models. In summary, by using a combination of phage and cell display, engineering of bispecificity into a 5 kDa small albumin-binding domain has been possible. To our knowledge, the obtained binders in this study are the smallest engineered bispecific proteins reported so far and are probably close to the size limitation in terms of obtaining two high affinity interacting surfaces on a single protein. Furthermore, the demonstrated approach should be applicable for generating bispecific affinity proteins to other interesting targets, providing potentially outstanding tissue penetration properties combined with a long *in vivo* half-life. Our data also suggest that since the two surfaces are discriminated from each other, the two affinities are independently tunable. The same target-specific binder could thus be optimized for both imaging applications, demanding short half-life, and therapeutics where long half-life is desired.
